# The power of data mining in diagnosis of childhood pneumonia

**DOI:** 10.1098/rsif.2016.0266

**Published:** 2016-07

**Authors:** Elina Naydenova, Athanasios Tsanas, Stephen Howie, Climent Casals-Pascual, Maarten De Vos

**Affiliations:** 1 Department of Engineering Science, Institute of Biomedical Engineering, University of Oxford, Oxford, UK; 2 Child Survival Theme, Medical Research Council Unit, Serrekunda, The Gambia; 3 Nuffield Department of Medicine, Wellcome Trust Centre for Human Genetics, University of Oxford, Oxford, UK

**Keywords:** childhood pneumonia, machine learning, diagnostics

## Abstract

Childhood pneumonia is the leading cause of death of children under the age of 5 years globally. Diagnostic information on the presence of infection, severity and aetiology (bacterial versus viral) is crucial for appropriate treatment. However, the derivation of such information requires advanced equipment (such as X-rays) and clinical expertise to correctly assess observational clinical signs (such as chest indrawing); both of these are often unavailable in resource-constrained settings. In this study, these challenges were addressed through the development of a suite of data mining tools, facilitating automated diagnosis through quantifiable features. Findings were validated on a large dataset comprising 780 children diagnosed with pneumonia and 801 age-matched healthy controls. Pneumonia was identified via four quantifiable vital signs (98.2% sensitivity and 97.6% specificity). Moreover, it was shown that severity can be determined through a combination of three vital signs and two lung sounds (72.4% sensitivity and 82.2% specificity); addition of a conventional biomarker (C-reactive protein) further improved severity predictions (89.1% sensitivity and 81.3% specificity). Finally, we demonstrated that aetiology can be determined using three vital signs and a newly proposed biomarker (lipocalin-2) (81.8% sensitivity and 90.6% specificity). These results suggest that a suite of carefully designed machine learning tools can be used to support multi-faceted diagnosis of childhood pneumonia in resource-constrained settings, compensating for the shortage of expensive equipment and highly trained clinicians.

## 1. Introduction

Pneumonia is the number one killer of children under the age of 5 years (more than 1.1 million deaths annually), causing more deaths than malaria, tuberculosis and HIV/AIDS combined [1–3]. More than 95% of the childhood pneumonia cases and 99% of subsequent deaths occur in developing countries [2]. Appropriate diagnostic assessment of childhood pneumonia typically relies on the use of advanced tools (such as X-rays and blood culture) as well as interpretation of observational diagnostic signs (chest indrawing and nasal flaring) by highly trained clinicians. Moreover, individual measurements are often insufficient and the clinical expert has to assess a combination of vital signs and other clinical characteristics for accurate diagnosis [4,5]. However, access to high-quality healthcare may often be limited in many low and middle income countries (LMICs) due to a shortage of appropriate medical equipment and clinical expertise.

Timely and accurate diagnosis that facilitates appropriate treatment has been reported to have the potential to reduce mortality by as much as 42% [3]. Most childhood pneumonia deaths are reported to occur in a relatively early stage of disease progression and complications can develop quickly. In resource-constrained settings, hospital facilities are often remote and community health workers (CHWs) need to differentiate between patients who can be managed locally and those in need of urgent referral. Thus, it is essential that *severity* can be determined as early and as accurately as possible in a point-of-care setting.

The World Health Organization (WHO) has developed a set of guidelines for diagnosis of childhood pneumonia in resource-constrained settings, directing health workers through identification of pneumonia and antibiotic prescription or hospitalization—the guidelines for integrated management of childhood illness (IMCI) [6]. However, a series of reports investigating the integration of these guidelines into clinical practice worldwide have reported reasonably high sensitivity of derived diagnosis (69–94%), overshadowed by poor specificity (16–67%) [7–9]. Consequently, unnecessary antibiotic prescription has risen, causing depletion of drug stocks and microbial resistance. Thus, it is essential that more specific (but equally sensitive) diagnostic tools are developed, and that objective measurements are used to reduce intra- and inter-user variability in diagnostic performance. Additionally, novel and affordable tools for determination of aetiology should be developed—currently, a combination of chest X-ray and blood culture are required for this.

Machine learning has been shown to be successful as a tool for strengthening diagnostic accuracy of hospitalized pneumonia patients: in particular, (i) identifying patients suitable for treatment at home and reducing healthcare costs [10,11] and (ii) predicting mortality in hospitalized patients [12,13]. These studies use a wide range of machine learning techniques, applied to datasets derived from electronic health records (EHR). EHR contain numerous variables acquired by experts using advanced diagnostic tools; it is unfeasible that such rich datasets could be regularly obtained in resource-constrained settings. Moreover, the focus of most of these studies is pneumonia in adults and yet manifestations of the disease in children are considerably different.

**Figure 1.**
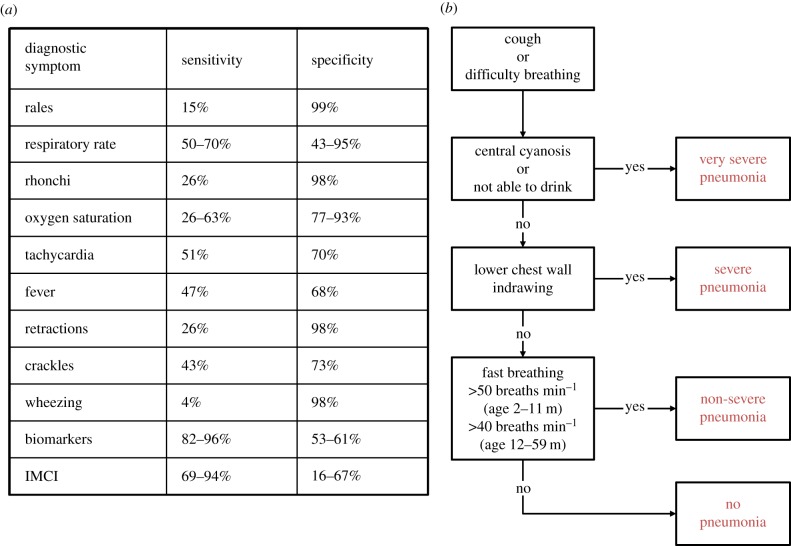
(*a*) Summary of sensitivity and specificity of individual clinical characteristics, derived from applying a threshold (as per standard clinical guidelines), as well as the integrated management of childhood illness (IMCI) guidelines [14–16]. (*b*) Diagnostic criteria used by a clinician to determine pneumonia outcome.

By contrast, research on the use of parsimonious datasets, comprising affordable point-of-care measurements for diagnostic support of childhood pneumonia is rare. Traditionally, basic analytical tools for thresholding individual variables have been used [14–16] but none of these variables have been found to be both sensitive and specific enough individually (figure 1). Abeyratne *et al*. reported the use of cough recordings, in combination with fever, deriving algorithms for automated detection of the cough sounds and subsequent identification of pneumonia. While this approach appears to deliver promising sensitivity (94%), specificity is lower (75%) and no information on severity or aetiology is derived. Additionally, the approach relies on continuous sound recording of the child in a hospital setting; in practice, consultation times are typically reported to be less than 2 min due to the large volume of patients in primary care facilities and the limited tolerance young children have for physical examinations [17].

Triaging systems based on data mining of a few basic vital signs have been investigated in the context of influenza and epidemic outbreaks in highly populous areas, delivering promising results (sensitivity and specificity of approximately 85%) [18,19]. We propose a suite of machine learning techniques for automated threefold diagnosis of childhood pneumonia (identification, severity and aetiology) based on variables that: (i) could be quantified unambiguously and (ii) have the potential to be measured affordably in resource-constrained settings. Such techniques could provide health workers with essential information and facilitate holistic evidence-based clinical decisions. In this study, each of the three diagnostic aspects was addressed separately, where (i) a minimal and most informative set of features was identified and (ii) machine learning algorithms were used to combine information from individual features and improve diagnosis in an automated way. The practical limitations of feature acquisition in a point-of-care setting were incorporated and the number of measurements needed during an examination was minimized. The analysis presented here builds upon a clinical study investigating the discovery of novel pneumonia-related biomarkers [20].

## 2. Data

The dataset analysed here was originally collected as part of a clinical study described by Huang *et al*. [20]. The 1581 participants were Gambian children aged 2–59 months. Various features were collected for each case. The full dataset consisted of 57 features (clinical characteristics), including measurable clinical variables (e.g. white blood cell count, neutrophils, haemoglobin, etc.), observational clinical characteristics (e.g. sleepiness, sternal indrawing, cough heard, etc.) and conventional vital signs (e.g. respiratory rate (RR), heart rate (HR), oxygen saturation (Osat), etc.). Additionally, selected cases also contained four biomarkers (C-reactive protein (CRP), lipocalin-2 (Lcn2), haptoglobin (Hap) and CD163 protein). We will demonstrate results on the following subsets:

— *Identification dataset* (1581 cases, 57 features). Seven hundred and eighty childhood pneumonia and 801 age- and gender-matched healthy controls were recruited.
— *First severity dataset* (780 cases, 57 features). From the 780 pneumonia cases, 458 had severe and 322 had non-severe pneumonia.
— *Second severity dataset* (180 cases, 61 features). One hundred and eighty of the pneumonia cases contained biomarker information—104 severe and 76 non-severe cases.
— *Aetiology dataset* (84 cases, 61 features). Only 84 cases had aetiology information, 22 bacterial and 62 viral, as gold standard diagnosis requires the acquisition of X-rays and blood culture.

The diagnostic outcome for each case was provided by a clinician, expanding on the WHO [21] and IMCI [6] guidelines. The criteria for identification of pneumonia and severity determination are listed in figure 1 and have been discussed in detail by Scott *et al*. in their search for a widely accepted clinical criteria for pneumonia classification [22]. Additionally, X-ray end consolidation and/or a positive blood culture result were used to differentiate bacterial from viral pneumonia cases. Further details on the data acquisition process can be found in the original clinical study [20].

## 3. Methodology

### 3.1. Preprocessing

Preprocessing of the original data was performed. The substantial number of missing values (up to 42% for some of the features) was addressed through imputation: features and cases containing less than 85% of the total number of entries were removed; the remaining missing values were imputed using feature median values (feature mean imputation was also tested but was observed to deliver equivalent results). Following standard statistical machine learning methodology, we extended the dimensionality of the original design matrix. Specifically, we introduced additional vectors for each feature that mirrored imputations—these vectors contained ‘ones’ where imputation was done and ‘zeros’ otherwise [23]. We will refer to these vectors as ‘ghost vectors’ throughout this study.

Several clinical features were excluded from the dataset as they are used in the clinical ‘gold standard’ and cannot be captured in a quantifiable way, e.g. ‘lower chest wall indrawing’. Details can be found in the feature list in the electronic supplementary material, §A.

In addition to imputation, all features were scaled to a similar range. For continuous valued features, this was achieved by subtracting their minimum value and dividing by their range of values. For discrete features, a vector of ones was added and the resulting sum was divided by the maximum values for the feature plus one, avoiding multiple zero entries. Gaussianizing of data via the Box–Cox transformation was also attempted but had no considerable effect on performance.

### 3.2. Feature selection

Feature selection in this study was driven by considerations related to data limitations and diagnostic application

— *Data considerations.* The curse of dimensionality has been shown to affect even powerful classifiers such as random forests (RFs) and support vector machines (SVMs) [24–26]. An exhaustive search over all possible feature combinations to determine the optimal feature subset is computationally intractable, hence we used a number of well-established feature selection approaches.
— *Diagnostic application considerations*. Point-of-care diagnostics would realistically afford a limited number of features. Therefore, a feature selection approach that took into account cost, acquisition time and quantifiability of measurements was used.

Seven feature selection techniques were used to investigate the predictive ability of features towards the outcome: maximum relevance on the basis of the linear (Pearson) correlation coefficient, maximum relevance minimum redundancy (mRMR), relief, Gram–Schmidt orthogonalization (GSO), least angle shrinkage and selection operator (LASSO), elastic net (EN) and sparse linear discriminant analysis (sLDA). A brief description of each technique is given in the electronic supplementary material.

**Figure 2.**
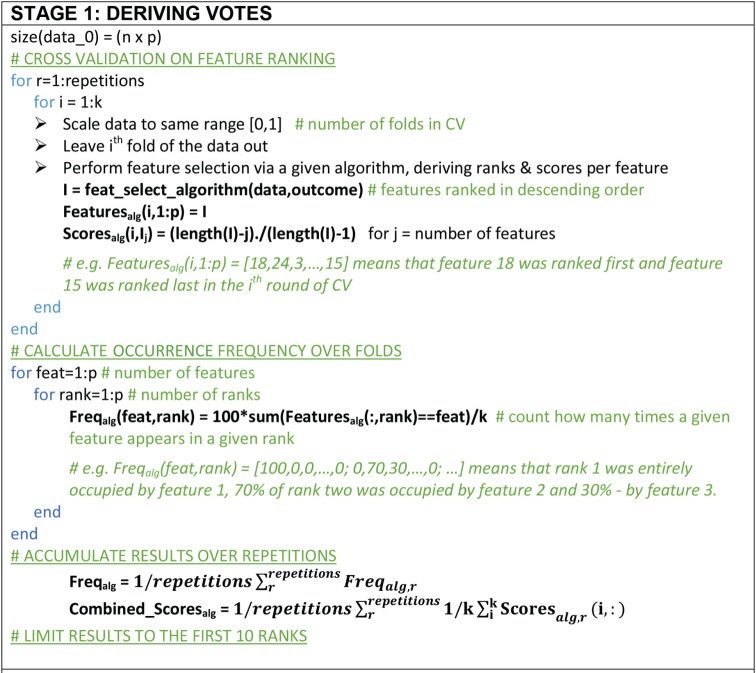
Pseudo-code for majority voting method for feature selection. We used 10-fold CV with 50 repetitions for statistical confidence.

A majority voting approach that consolidates results from all techniques was developed in order to dilute the bias of individual techniques and obtain a more objective selection of features. Using 10-fold cross-validation and 50 repetitions, feature selection was performed on nine tenths of the data via each one of the seven techniques, after all features were scaled to the same range. The frequency with which each feature occupied each rank was calculated across folds and results were averaged across the number of repetitions. Additionally, normalized scores were derived to reflect the overall frequency of ranks. This approach is summarized in the pseudo-code figure 2. Next, two inclusion conditions were applied. First, a feature should appear in the top 10 of at least three ‘fundamentally different’ techniques; the pairs (correlation and mRMR) and (LASSO and EN) share conceptually similar theoretical bases and are not ‘fundamentally different’. Second, the feature should be measurable in a point-of-care setting in an affordable way. The scores associated with selected features were used to derive a feature ranking. The SpaSM toolbox was used to perform LASSO, EN and sLDA in Matlab [27]; we used our own custom-based implementations for the remaining feature selection algorithms.

### 3.3. Classification

The IMCI guidelines are widely used by CHWs in low-resource settings to identify children at risk of pneumonia. In this paper, we compare a number of machine learning techniques with the potential to improve performance of the IMCI guidelines. Historically, logistic regression (LR) has been the most popular technique in classification contexts in the medical domain, including assessment of pneumonia. In this study, LR was used as a benchmark technique and the applicability of two other classifiers, SVMs and RFs, was investigated. The application of all three techniques to various medical diagnostics problems has been well-documented in the literature [28,29] and a brief overview of the underlying principles is given in the electronic supplementary material.

### 3.4. Performance generalization

The generalization of each machine learning algorithm (i.e. its expected performance on unseen data) was assessed using either fourfold cross-validation (for the bigger data subsets) or leave-one-out (for the smaller data subsets). For each training set, preprocessing as described in §3.1 was performed; these operations were then applied to the test set, using characteristic parameters derived from the training set. Additionally, internal fivefold cross-validation was performed to optimize classifier parameters in each training set, based on area under the curve (AUC).

Using the feature ranking, an increasing number of features were gradually fed into the classifier, with a separate algorithm trained for each number. The following performance metrics were recorded from the test set: sensitivity, specificity, AUC, balanced accuracy and Matthew's correlation coefficient (MCC), where the latter two were defined as: balanced accuracy = 0*.*5 (sensitivity + specificity); MCC = (TP × TN − FP × FN)*/*√(TP + FP)(TP + FN)(TN + FP)(TN + FN), where TP = true positive, TN = true negative, FP = false positive and FN = false negative. MCC and balanced accuracy were used to account for the disproportionality of outcomes, particularly evident in the aetiology dataset.

In a thorough validation approach, the steps above were repeated 20 times to offset any bias in the split of the data into train and test, and assess performance variance (which ideally will be low in order to have confidence in the reported errors). Algorithm performance in this paper is reported in terms of mean values and variance across the repetitions. The performance of the three classifiers was assessed, identifying both the optimal number of features as well as the best performing algorithm.

### 3.5. Visualization for interpretation

Childhood pneumonia presents through a complex inter-action of symptoms. Therefore, visualization of the identified diagnostic features was necessary to link this data-driven approach back to the clinical rationale. A dimensionality reduction technique called t-stochastic neighbourhood embedding (t-SNE) was applied [30]. Medical data, such as the pneumonia data used in this study, occupies nonlinear manifolds; consequently, linear methods such as principal component analysis (PCA) [31] and multidimensionality scaling (MDS) [32] are insufficient as they mainly preserve the separation between dissimilar data entries within a low-dimensional space, at the expense of closure information concerning similar entries. t-SNE has been previously reported to capture aspects of both the local as well as the global structure, preserving the neighbouring probabilities of samples. It calculates Euclidean distances between data entries and derives similarities (conditional probabilities) by assuming a Student-*t* distribution [30]. In this study, t-SNE was used for projecting the higher dimensional feature space onto a two-dimensional space.

## 4. Results

Results for each of the three diagnostic challenges are presented separately in §4. Additionally, the electronic supplementary material, §D, contains a description of various machine learning techniques that were applied to this problem but led to worse results than those reported here. Nevertheless, these lessons can be of value to the research community and others working on this specific problem.

### 4.1. Disease identification

Applying the feature selection methodology outlined previously, the following feature subset was selected: RR, HR, temperature (T), malnutrition (WHZ) and Osat, listed in descending order of importance. A full list of the selected features can be found in the electronic supplementary material, figure S1. RR, HR, T and Osat had 13 missing values each, WHZ had 22. Values were imputed using the procedure described in §3.1., leading to the creation of five ghost vectors.

We experimented with different loss function approaches (balanced accuracy, MCC) for the three classifiers tested (LR, SVM, RF). The MCC was observed to be most favourable, optimizing performance in the test set as a result of fine-tuning of model parameters in the training/validation set. Specifically, for SVM, a Gaussian radial basis function kernel, with a kernel width, *γ*, of 0.1 and a cost parameter of 1000 were found to deliver the best performance; for RF, 750 decision trees and searching over two variables at each tree node were the optimal hyperparameters.

**Figure 3.**
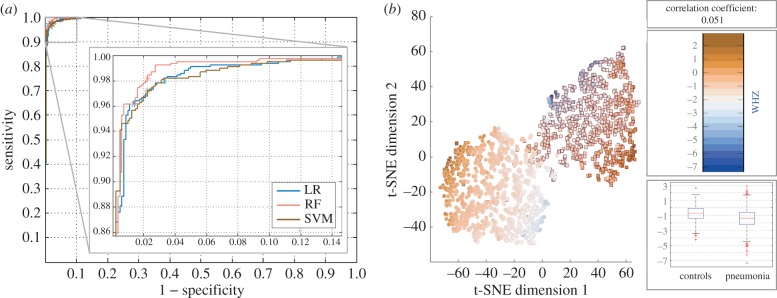
(*a*) Receiver operating characteristic (ROC) curve for the three classifier applied to the problem of identifying pneumonia; the location of the test results on the ROC curve as per the MCC optimization are denoted via an ‘asterisk’. (*b*) Distribution of malnutrition values (measured via the WHO *Z*-score) across pneumonia (square) cases and controls (circle); t-SNE has been applied to all five clinical characteristics in order to reduce dimensionality to two dimensions, leading to the creation of two mostly independent ‘clusters’. The colour scale represents the corresponding *Z*-score per entry. The boxes on the right contain the following information: (top) correlation coefficient between the malnutrition feature and outcome; (middle) colour bar for the colour scale used in the plot; (bottom) a boxplot of the malnutrition data, split between the two classes. In the latter, the central/red line represents the median, the edges are the 25th and the 75th percentiles, and the dashed lines extend to the most extreme data points.

The three classification techniques exhibited comparable performance, with somewhat more favourable sensitivity and specificity obtained with RF (figure 3*a*). LR was seen to sometimes outperform SVM but exhibited larger variance. The addition of the ghost vectors had a very limited effect on classification performance, with changes in all metrics limited to ±1%. Taking a closer look at the RF algorithm, four features (RR, HR, Osat and T), with the addition of age, delivered maximal results: 98.2% (95% CI 97.9–98.8%) sensitivity; 97.6% (95% CI 97.1–98.0%) specificity; 99.7% (95% CI 99.3–99.8%) AUC; 95.9% (95% CI 95.3–96.5%) MCC. From the cases that were falsely classified as controls, 33% were severe pneumonia. Moreover, reducing the number of features to three, worsened performance marginally: 98.2% (95% CI 97.8–98.6%) sensitivity; 97.1% (95% CI 96.8–97.5%) specificity; 99.6% (95% CI 99.5–99.6%) AUC; 95.2% (95% CI 94.9–96.1%) MCC. Somewhat surprisingly, the addition of malnutrition did not improve results. The distribution of malnutrition values across the two classes (pneumonia and controls) was further examined on the basis of t-SNE dimensionality reduction (figure 3*b*).

### 4.2. Severity determination

The aim of this part of the analysis was to investigate whether it is possible to predict severity using quantifiable features rather than the observational features included in the ‘gold standard’ guidelines such as ‘chest wall indrawing’. Additionally, it was also investigated whether biomarkers could be fused with vital signs to improve severity prediction. For the purposes of an early warning algorithm, severity was divided into ‘non-severe’ and ‘severe’, with the latter combining both severe and very severe pneumonia cases.

In the first severity dataset, selected features were: RR, Osat, crackles, grunting, HR. In the second severity dataset, selected features were: RR, grunting, crackles, CRP, HR, Osat, CD163, Hap, Lcn2. A full list of the selected features is available in the electronic supplementary material, figure S2. In the first severity dataset, all five features had less than three missing values each, introducing five ghost vectors. In the second severity dataset, the clinical signs had no missing values and the missing values among the biomarkers were less than 8% (after exclusion of 19 cases that were missing seven out of nine features), introducing four ghost vectors.

**Figure 4.**
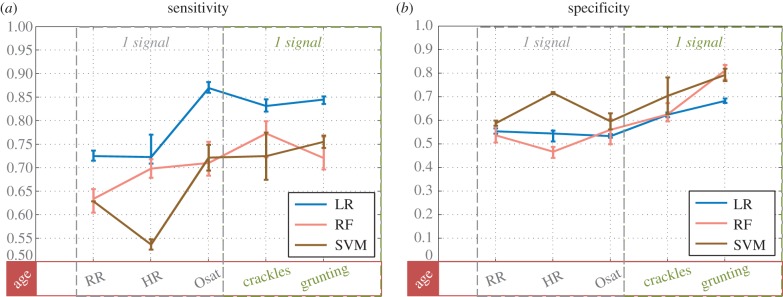
Classifiers' performance in determining severity, reported as sensitivity (*a*) and specificity (*b*) for an increasing number of features. Along the *x*-axis, features are listed in an additive manner, i.e. each x-entry represents classification with that specific feature and all features on the left of it. Additionally, age was added to the classifier throughout. Values represent the mean as well as the minimum and maximum values attained across the multiple iterations. Note that the scale of the *y*-axis is different in each plot. Features that can be derived from the same measurement/signal have been listed adjacently and surrounded by a dashed box.

**Figure 5.**
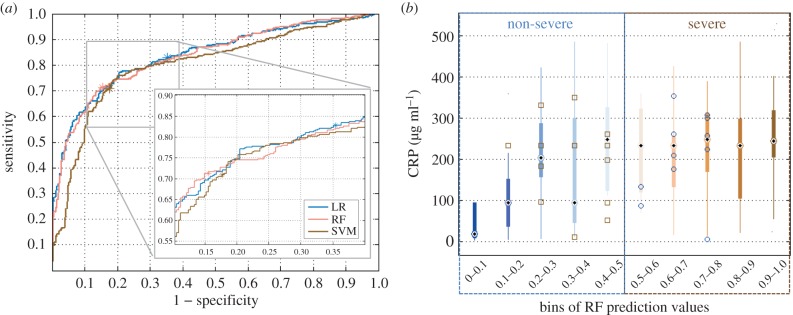
(*a*) Receiver operating characteristic (ROC) curve for the three classifier applied to the problem of determining severity; the location of the test results on the ROC curve as per the MCC optimization are denoted via an asterisk. (*b*) Distribution of CRP values across 10 probabilistic groups/bins. Along the *x*-axis, the range of RF probabilistic predictions was divided into 10 bins, where bin 1 contains cases assigned probabilities between [0,0.1], i.e. 90–100% certainty of non-severe pneumonia, and bin 10 contains cases assigned probabilities between [0.9,1], i.e. 90–100% certainty of severe pneumonia. The number of predicted cases in bins 1–10 were: 29, 21, 14, 23, 12, 7, 19, 23, 23, 28. In each bin, the feature distribution of correctly classified cases is visualized via a boxplot. In each box, the central dot represents the median, the edges are the 25th and the 75th percentiles, and the thin lines extend to the most extreme data points. Misclassified cases in each bin are plotted on top of the boxplot with squares denoting severe cases and circles denoting non-severe ones.

First, the mixture of vital signs and lung sounds, listed for the first severity dataset, was used to classify severity (figure 4). The following hyperparameters were found to lead to best performance: a Gaussian radial basis function kernel, with a kernel with *γ* of 0.01 and a cost parameter of 10 000 for SVM; 750 decision trees and searching over two variables at each tree node for RF. MCC proved to be the most suitable optimization metric. Some differences were observed between the classifiers: RF and SVM performed comparably, whereas LR achieved better sensitivity at the cost of specificity (figures 4 and 5). The addition of the ghost vectors had very limited effect on classification, with changes in all metrics limited to ±0*.*8%. With five features (and age): LR delivered sensitivity of 84.6% (95% CI 83.6–85.2%); specificity of 68.3% (95% CI 67.5–69.3%); AUC of 83.7% (95% CI 83.2–83.8%); MCC of 53.6% (95% CI 52.1–55.2%); RF delivered sensitivity of 71.8% (95% CI 70.0%–76.6%); specificity of 81.8% (95% CI 73.3–83.5%); AUC of 83.5% (95% CI 82.5–83.8%); MCC of 52.9% (95% CI 49.6–55.2%).

The five clinical features listed above could be extracted via two measurements, keeping cost down, while also ensuring feasibility. Biomarkers could be used to improve performance but are associated with additional cost (both for the development of the assay, if it does not exist, as well as the cost of individual probes). Consequently, when introducing biomarkers to the feature set we attempted to keep their number as low as possible (one or two) and identify optimal combinations (table 1). CRP and Hap added to the three vital signs and two lung sounds, were seen to deliver best results: sensitivity of 91.4% (95% CI 89.4%–92.3%); specificity of 83.2% (95% CI 82.1–84.2%); AUC of 94.2% (95% CI 93.7–94.3%); MCC of 73.9% (95% CI 72.8–77.0%). However, with just one biomarker (CRP) and the same list of remaining features, performance was not much worse: sensitivity of 88.5% (95% CI 87.5%–90.4%); specificity of 82.1% (95% CI 80.0–84.2%); AUC of 92.3% (95% CI 92.0–94.9%); MCC of 71.8% (95% CI 68.9–72.9%). The reported results were obtained with RF; with more than five features, LR's specificity fell below 60% and computational time with SVM was very long (17 h for the full validation routine, compared with 2 h with RF).

**Table 1.** Sensitivity and specificity of severity determination. First, biomarkers are added individually, followed by combinations of pairs of the top three performing biomarkers. With five features, LR outperformed RF's sensitivity; with more features, RF delivered both greater sensitivity and specificity.

no. features	features list	sensitivity	specificity
5^a^	RR, HR, Osat, crackles, grunting	0.85	0.68
5^b^	RR, HR, Osat, crackles, grunting	0.72	0.82
6^b^	RR, HR, Osat, crackles, grunting + CRP	0.89	0.82
6^b^	RR, HR, Osat, crackles, grunting + Lcn2	0.87	0.8
6^b^	RR, HR, Osat, crackles, grunting + Hap	0.88	0.79
6^b^	RR, HR, Osat, crackles, grunting + CD163	0.88	0.77
7^b^	RR, HR, Osat, crackles, grunting + CRP + Lcn2	0.91	0.78
7^b^	RR, HR, Osat, crackles, grunting + CRP + Hap	0.91	0.83
7^b^	RR, HR, Osat, crackles, grunting + Lcn2 + Hap	0.88	0.76

^a^Classification via LR.

^b^Classification via RF.

Taking a closer look at the classifier's predictions, most cases from both classes were classified with high level of certainty. To trace the roots of any uncertainty, the probabilistic outcomes were split into 10 bins and the distribution of individual features in each bin was investigated (CRP in figure 5 and RR in the electronic supplementary material, figure S4). From this, bins 1 and 2, and, 9 and 10 contained only one misclassified case and displayed a substantial difference between their CRP and RR values. Cases with more moderate CRP and RR values led to more uncertainty and consequently higher misclassification rates.

Finally, the IMCI severity guidelines were used for comparison, delivering 79.3% sensitivity and 67.7% specificity (first severity dataset).

### 4.3. Aetiology determination

This part of the study explored whether a combination of vital signs and biomarkers could be used to predict aetiology (bacterial versus viral), providing a potential alternative in settings where X-rays and blood culture laboratories are unavailable.

The feature selection identified six features: Lcn2, Hap, RR, CRP, HR, CD163; a full list of selected features is available in the electronic supplementary material, figure S3. Missing values were imputed using the median value of features, where imputation was under 10% in all concerned features, leading to the creation of four ghost vectors.

**Figure 6.**
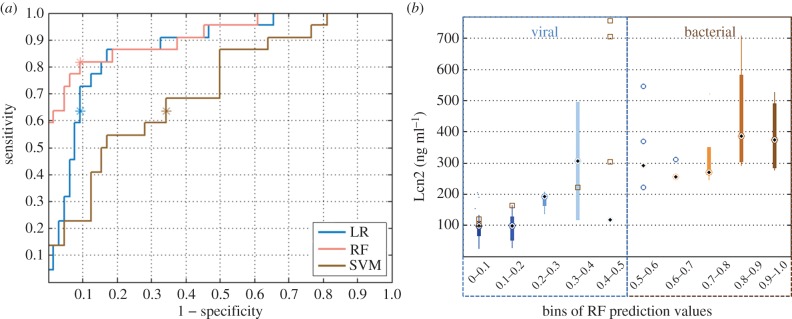
(*a*) Receiver operating characteristic (ROC) curve for the three classifier applied to the problem of aetiology determination; the location of the test results on the ROC curve as per the MCC optimization are denoted via an ‘asterisk’. (*b*) Distribution of Lcn2 values across 10 probabilistic bins, presented as in figure 5. The number of predicted cases in bins 1–10 were: 42, 14, 4, 3, 4, 4, 2, 5, 4, 7. Misclassified cases in each bin are plotted on top of the boxplot with squares denoting bacterial and circles denoting viral cases.

Optimization of hyperparameters was performed via MCC, selecting a *γ* of 0.01 and a cost parameter of 1000; and 750 decision trees and searching over three variables at each tree node for RF. Substantial differences were observed in the performance of the three classifiers (figure 6*a*)—SVM was seen to underperform despite efforts to fine-tune the two relevant hyperparameters. RF was seen to deliver better sensitivity than LR for a comparable specificity. Similarly to the severity part of the study, biomarkers were added to the feature set, aiming to minimize the number required to achieve satisfactory classification results (table 2). The addition of Lcn2 to RR, HR and Osat was seen to deliver good performance: sensitivity of 81.8% (95% CI 81.8–81.8%); specificity of 90.6% (95% CI 89.1–92.2%); AUC of 91.6% (95% CI 89.6–92.8%); MCC of 70.5% (95% CI 68.1–73.0%). Adding a second biomarker did not improve results. The addition of ghost vectors led to marginal changes in performance (±0*.*7%).

**Table 2.** Sensitivity and specificity of RF for prediction of aetiology. The best performing biomarker was identified first; combinations with a second biomarker were explored with no substantial change in performance.

no. features	features list	sensitivity	specificity
3	RR, HR, Osat	0.50	0.86
4	RR, HR, Osat + CRP	0.64	0.88
4	RR, HR, Osat + Lcn2	0.82	0.91
4	RR, HR, Osat + Hap	0.64	0.86
4	RR, HR, Osat + CD163	0.46	0.91
5	RR, HR, Osat + Lcn2 + CRP	0.82	0.88
5	RR, HR, Osat + Lcn2 + Hap	0.82	0.91
5	RR, HR, Osat + Lcn2 + CD163	0.82	0.89

Similar to the analysis for severity, probabilistic predictions were split into bins and the distribution of individual features across these bins was studied (figure 6*b*). Cases with Lcn2 values below 200 ng ml^−1^, were classified as viral with a high degree of certainty. However, three bacterial cases were seen to have Lcn2 values in that range and some viral cases also presented with elevated Lcn2. Additionally, the relationship between severity and aetiology of pneumonia is not straight-forward. The available dataset contained a mixture of cases spread across the severity and aetiology categories: from the bacterial cases, 10 were severe and 12 non-severe; from the viral cases, 12 were severe and 52 non-severe. Three of the severe cases (13.6%) were misclassified in terms of their aetiology. Based on the IMCI guidelines, all 86 cases should have been administered antibiotics. Hence, according to the IMCI guidelines, 100% of the viral cases would have been misclassified according to their aetiology.

## 5. Discussion and conclusion

Childhood pneumonia presents a severe global health challenge, costing the lives of more than a million young children annually. Affordable and timely diagnostics that facilitate appropriate treatment could help save many of these lives. However, existing diagnostic approaches are often inappropriate for resource-constrained settings and novel techniques are required to leapfrog the gap in medical expertise and expensive equipment. Data mining has the potential to offer such a diagnostic remedy, if utilised in a clinically driven and user-centric manner. This study investigated the use of a suite of machine learning techniques to derive clinical insights regarding a holistic diagnostic assessment, including identification, severity and aetiology determination. The proposed approach applied to eight features (four vital signs (RR, HR, Osat, T), two lung sounds (grunting, crackles) and two biomarkers (CRP, Lcn2)) could: identify pneumonia with 98.2% sensitivity and 97.6% specificity; determine severity with 89.1% sensitivity and 81.2% specificity; and determine aetiology with 81.8% sensitivity and 90.6% specificity. These findings are very clinically relevant as they suggest that machine learning could be used to strengthen the capacity of health system in low-resource settings to deal with childhood pneumonia, despite sparsity of advanced equipment and highly trained clinicians.

The purpose of the analysis was not to simply automate the guidelines but rather to offer a more accurate and reproducible alternative. To achieve this, some of the features highlighted by the feature selection techniques as predictive (‘cough’, ‘days unwell’, ‘head nodding’) were excluded because they could not be measured unambiguously. Both ‘cough’ and ‘days unwell’ rely on a parent's careful observations. Similarly, features containing a huge degree of variability between providers of health (e.g. head nodding) were also excluded. However, lung sounds (‘crackles’, ‘grunting’, ‘bronchial breathing’) were kept in the feature set as recent technological advancements have indicated that the acquisition of lung sounds could be automated through appropriate signal processing.

Some of the selected features were grouped together, e.g. RR, HR and Osat in figure 4, regardless of the ranking order derived during feature selection. This was done in order to illustrate that some features could be obtained through the same measurement/signal, reducing complexity of clinical examination and cost. For example, the PPG signal obtained through a pulse oximeter could be used to derive RR, HR and Osat. Similarly, multiple lung sounds (crackles, grunting, bronchial breathing) can be obtained through a single stethoscope measurement.

From the classifiers examined, RF was seen to outperform other methods (including LR which is conventionally used in clinical studies), indicating likely nonlinear interaction between the clinical signs measured. Identification of pneumonia was achieved with a high degree of confidence using four clinical features that could be derived from just two measurements—a PPG measurement (delivering RR, HR and Osat) and a temperature measurement. However, the dataset analysed in this study contained some limitations. Specifically, the control cases were generally quite healthy (apart from some odd cases of elevated HR and low SpO2). In a realistic clinical setting children will also present with various other diseases; therefore, a reliable evidence-based machine learning algorithm should ideally be trained to differentiate childhood pneumonia from other conditions that might appear similar (e.g. malaria or tuberculosis).

The severity analysis elucidated a few key findings. First, with three vital signs and two lung sounds, it was possible to determine severity with high specificity (82.2%) but lower sensitivity (72.4%) using an RF algorithm. However, keeping the diagnostic application in mind, low sensitivity would mean severely ill children who should have been referred to hospital get missed. The use of LR had the opposite effect, favouring sensitivity at the expense of specificity (figure 4). Consequently, triaging of severe cases using the LR algorithm might be more efficient but would also lead to referral of a lot of non-severe cases. Future work could consider combining the two algorithms to defuse some of this uncertainty. Second, the addition of biomarkers was seen to only improve sensitivity, with very limited changes to specificity (table 1). With or without biomarker information, fusion of features via machine learning was seen to outperform the IMCI guidelines for severity (as demonstrated in §4.2.), where the latter uses observational and unquantifiable features.

Finally, the study also suggested an alternative source of aetiology information, which is typically obtained using X-rays and blood culture, by combining a couple of vital signs with a recently proposed biomarker (Lcn2). Moreover, only three out of the 22 severe cases were misclassified (13.6%) in terms of their aetiology. Misclassifications within the aetiology problem would be most detrimental in cases of severe bacterial/viral pneumonia as this could hinder the timely administration of appropriate treatment. This information could be crucial in settings where access to advanced medical technologies is limited, provided a point-of-care test for Lcn2 is developed. In order to reliably validate the ability of this approach to replace the use of X-rays and blood culture, especially in settings where these are not available, a bigger dataset would be required.

Multiple risk factors associated with pneumonia have been identified in the literature, with malnutrition playing a substantial role in the clinical outcome. Specifically, malnutrition has been quoted as an underlying factor in 35% of deaths in children under 5-years old, including those from pneumonia [2]. Pneumonia in severely malnourished children is often undetected, in the absence of advanced imaging, leading to high mortality. In the dataset analysed here, extreme malnutrition scores (from figure 3*b*, scores less than −4 and more than 1) were seen to be related to the presence of pneumonia, confirming the status of malnutrition as a high risk factor. However, moderate malnutrition scores were seen to be equally distributed across both pneumonia and controls. Additionally, malnutrition showed limited significance to prediction of severity or aetiology. Nevertheless, it is expected that malnutrition would be more relevant as a predictor of survival but the dataset available did not contain information on such outcomes.

Biomarkers were included in the analysis despite the fact that affordable point-of-care tools might not be commercially available for all of them yet. Nevertheless, research in this area has delivered promising results. For example, Martinez *et al*. reported a production price of US$0.01 for a paper-based analytical device and multiple applications for this type of technology have been explored [33]. Point-of-care assays for CRP have been developed by several commercial providers. Lcn2 cannot be currently measured in a point-of-care setting. However, the results obtained in this study highlighted that CRP and Lcn2 could facilitate both severity and aetiology determination, supporting the need for the development of affordable point-of-care assays for both biomarkers.

This study provides a theoretical foundation upon which the research team will be looking to expand both in terms of the analysis of larger and richer datasets as well as the design of appropriate point-of-care tools to be used for acquisition of some of the key parameters (e.g. detection algorithms for lung sounds via a low-cost digital stethoscope). Hence, a mobile application connected to low-cost diagnostic tools (a pulse oximeter and digital stethoscope) has been designed, with the user interface designed for basically trained CHWs. As a next step, it is crucial to validate findings on a dataset obtained in a community setting, where initial triaging for pneumonia would take place. For this purpose, the research team is designing a study that will collect data via the described tools in the community (e.g. in an urban slum), with outcomes validated at a public hospital. Consequently, this will allow validation of the proposed machine learning approach, as well as the development of additional algorithms to (i) differentiate pneumonia from other childhood diseases, (ii) stratify severity based on disease evolution, and (iii) predict potential complications early.

## Supplementary Material

Supplementary Material

## Data accessibility

The data supporting this article have been registered on Oxford University Research Archive (ORA) and can be accessed via http://dx.doi.org/10.5287/bodleian:ht24wj41z.
