# Supplementary material for: The power of data mining in diagnosis of childhood pneumonia
Source: J R Soc Interface. 2016 Jul;13(120):20160266. doi: 10.1098/rsif.2016.0266 (PMC4971218; doi:10.1098/rsif.2016.0266)
Supplement: Supplementary Material [file rsif20160266supp1.doc]

Supplementary material

1. *Data*

Table I

Full list of clinical features recorded during study


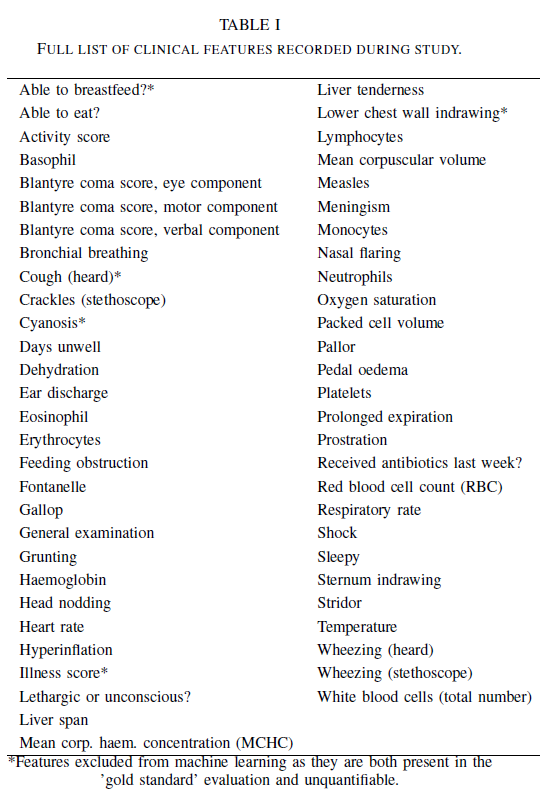


1. *Methodology*

1) *Feature selection techniques*: A brief description of each of the seven techniques used for feature selection: maximum relevance on the basis of the linear (Pearson) correlation coefficient, maximum Relevance Minimum Redundancy (mRMR), Relief, Gram-Schmidt Orthogonalisation (GSO), Least Angle Shrinkage and Selection Operator (LASSO), Elastic Net (EN) and sparse Linear Discriminant Analysis (sLDA). For all, **X** is a dataset of *m* features and *n* cases, *i* and *j* denote the feature and case indices respectively, and **y** is the outcome:

- *Correlation*

$\mathrm{corr}\left( \mathbf{x}_{i},\mathbf{y} \right)= cov\left( \mathbf{x}_{i},\mathbf{y} \right)/\sqrt{\mathrm{var}\left( \mathbf{x}_{i} \right)*var(\mathbf{y})}$ (1)

- *mRMR*: The technique first originated from the derivation of mutual information between a feature and the outcome to quantify relevance:

$I\left( \mathbf{x}_{i},\mathbf{y} \right)= \int\int p\left( \mathbf{x}_{i},\mathbf{y} \right)*log\frac{p\left( \mathbf{x}_{i},\mathbf{y} \right)}{p\left( \mathbf{x}_{i} \right)*p(\mathbf{y})}dx_{i}\mathrm{dy}$ (2)

Next, the concept of redundancy was added by computing the mutual information between pairs of features. Therefore, mRMR looks for a compromise between relevance and redundancy:

$mRMR= \max_{i\in Q-S} [I\left( \mathbf{x}_{i},\mathbf{y} \right)-\frac{1}{|S|}\sum_{s\in S} \mathbf{I}(\mathbf{x}_{i},\mathbf{x}_{\mathbf{s}})]$ (3)

where Q contains the indices of all features from the original feature space, **x**_s_ is a feature selected from the subspace of selected features S and |S| is its cardinality. In this study, we used a computationally fast version of mRMR that uses Spearman coefficients rather than mutual information [1].

- *Relief*: This is feature weighting algorithm that makes use of the concept of *Nearest Hit (NH)* and *Nearest Miss (NM)*, where the weight associated with each feature ($b_{i}$) is derived from:

$b_{i}= \frac{1}{q}\sum_{i=1}^{q} \left( -\frac{1}{\left| NH\left( \mathbf{x}_{j} \right) \right|}*\sum_{\mathbf{x}_{k}\in NH\left( \mathbf{x}_{j} \right)} \left| \left| x_{i,j}-x_{k,i} \right| \right|+\sum_{\mathbf{y}_{l}\neq\mathbf{y}_{j}} \frac{1}{NM(\mathbf{x}_{j})}*\frac{p(\mathbf{y}=y_{l})}{1-p(\mathbf{y}=y_{j})}\sum_{\mathbf{x}_{k}\in NM(\mathbf{x}_{j})} |\left| x_{i,j}-x_{k,j} \right|| \right)$

(4)

where q represents the number of randomly samples cases (*q*=*n* for an exhaustive search), |.| denotes the size of NH and NM and ||.|| is a distance metric (Euclidian distance).

- *GSO*: This technique uses orthogonal decomposition of features to evaluate and select them independently. To do this, the feature which best explains the outcome is elected by maximising:

$\cos^{2}\left( \mathbf{x}_{i},\mathbf{y} \right)= \frac{{(\mathbf{x}_{i}*\mathbf{y})}^{2}}{{|\left| \mathbf{x}_{i} \right||}^{2}{|\left| \mathbf{y} \right||}^{2}}$ (5)

Subsequently, overlapping information is discarded from both the outcome and the remaining features by projecting them onto the null subspace; hence: $\mathbf{x}_{i,new}= \mathbf{x}_{i}-\mathrm{proj}_{w1}(\mathbf{x}_{i})$, where w1 is the space spanned by y and proj($\mathbf{x}_{i})$ [2].

- *LASSO:* This technique uses the L_1_-norm as a sparsity promoting function to both minimise the prediction error and reduce the number of features, in a classical regression setting [3]. This is achieved through:

$\mathbf{b}_{LASSO}=arg\min_{b} \sum_{j=1}^{n} {(y_{i}-\sum_{j=1}^{m} x_{i,j}b_{i})}^{2}+\lambda\sum_{i=1}^{m} |b_{i}|$

(6)

where **b** = (*b_1_,…,b_m_*) represents the ordinary least squares parameters and λ is a regularisation parameter controlling shrinkage.

- *EN:* Building upon LASSO, EN can identify groups of variables and not select one feature arbitrarily from a set of similar features. The L2-norm penalty is added to group variables, coupled with a second regularisation parameter (λ_2_) [4]:

$\mathbf{b}_{EN}=arg\min_{b} \sum_{j=1}^{n} {(\mathbf{y}_{i}-\sum_{i=1}^{m} x_{ji}b_{i})}^{2}+\lambda_{2}\sum_{i=1}^{m} {|b_{i}|}^{2}+\lambda_{1}\sum_{i=1}^{m} |b_{i}|$

$$(7)$$

- *sLDA:* Linear Discriminant Analysis identifies a low-dimensional space that maximises the separation of the classes when the data is projected ($\mathbf{X}_{new}= \mathbf{w}^{T}*\mathbf{X}$). Consequently, the technique looks for an optimal **w** that maximises $J\left( w \right)= \frac{{(\tilde{m_{1}}-\tilde{m_{2}})}^{2}}{{\tilde{s_{1}}}^{2}+{\tilde{s_{2}}}^{2}} ,$where $\tilde{m_{1}}$ and $\tilde{m_{2}}$ are the multidimensional sample means of projected data, and $\tilde{s_{1}}$ and $\tilde{s_{2}}$ are the scatter parameters for projected samples, in the two classes for a given choice of w. A sparseness criterion using the elastic net penalty, as defined by Clemmensen et al., was added [5].

2) *Classification techniques*: An overview of the classification techniques used, where the definitions of variables from the feature selection discussion above have been preserved:

- *LR:* A widely used linear classifier, LR derives outcome predictions via the sigmoid function:

$p\left( \mathbf{x}_{j},a,\mathbf{b} \right)=1/(1+{exp}^{-(a+\boldsymbol{b}*\mathbf{x}_{\mathbf{j}})})$ (8)

where *a* + **b*****x***_j_* = 0 defines the decision boundary

separating the classes. The *a* and **b** parameters were


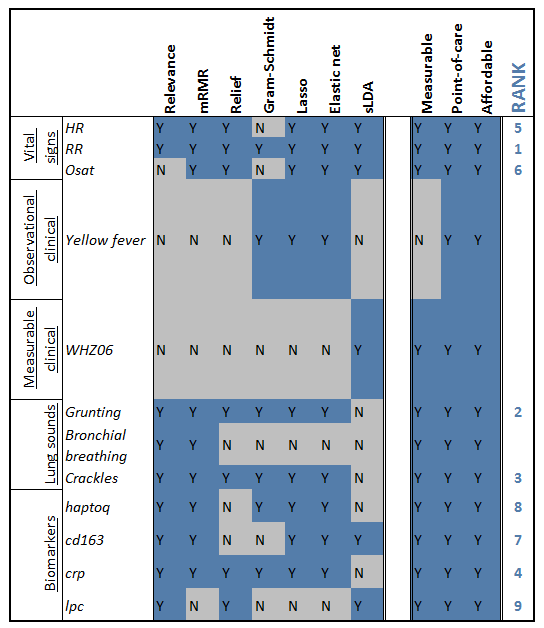
optimised in this study through a validation approach described in the main body of the paper.

- *SVM*: The principle behind SVM is the identification of a hyperplane (f(**X**)) that creates boundaries in the feature space to differentiate the classes. In the case of linearly separable data, the hyperplane is given by:

$f\left( \mathbf{X} \right)= \sum_{i=1}^{m} b_{i}\mathbf{x}_{i}+a=0$ (9)

In the case of non-linearly separable problems, a kernel function is applied to map the data to a high-dimensional feature space before constructing the linear decision function. The LIBSVM implementation [6] was used to optimise a Gaussian radial basis function kernel, determining the optimal values of the kernel width γ and the penalty parameter C though a grid search of possible values.

- *RF*: Random forests is a non-parametric technique formed from multiple simple base learners (i.e. trees). The method of Classification and Regression Tree (CART) partitions the feature space into sub-regions by identifying splits (”nodes”), consequently learning an increasingly detailed mapping between the data and the outcome [7]. Majority voting from multiple base learners is used to construct ensembles, diluting any bias in the classification of samples [8]. The number of trees and the number of features for splitting were optimised though a grid search of possible values - [50,150,250,350,450,550] and [1,…,f] respectively, where f is the number of features and the latter range was incremented by 1.

1. *Results*

Results, complementary to those reported in the main

body of the paper, are reported in Figures 1, 2, 3 and 4.


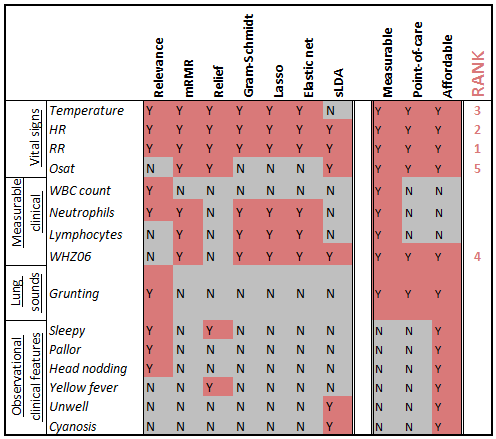


Fig. 1. Identification of pneumonia - selected features in descending order of their scores: Respiratory Rate (RR), Heart Rate (HR), Temperature (T), Malnutrition (measured via the WHO Z-score - WHZ) and Oxygen Saturation (Osat).

Fig. 2. Severity determination - selected features in descending order of their scores based on the Second Severity dataset: RR, Grunting, Crackles, CRP, HR, Osat, CD163, Haptoglobin (haptog), Lipocalin-2 (Lcn2).

1. *Further investigations*

A number of other techniques were applied to the dataset with limited success. Nevertheless, such lessons are valuable for the research community and are therefore summarised here.

1. *Pre-processing*: The effects of discretisation of continuous features were investigated using the Class-Attribute Interdependence Maximization (CAIM) algorithm [9]. Discretisation was performed in other studies mixing continuous and discrete features [10], [11]; however, the classifiers used in this study were capable of dealing with the mixture of features and hence experienced no improvement through the addition of CAIM.


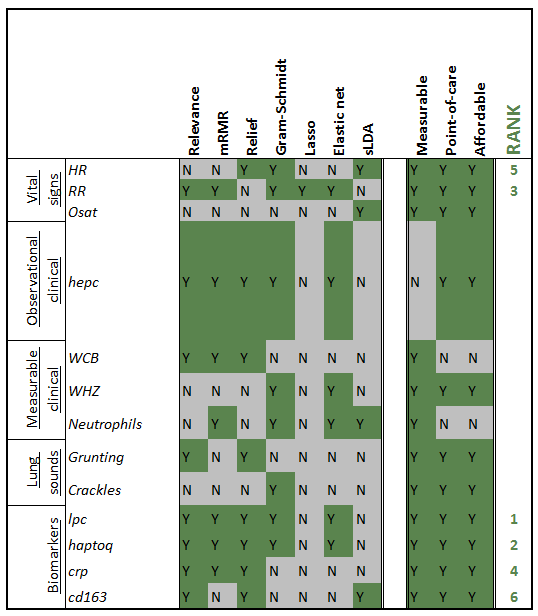


Fig. 3. Aetiology determination - selected features in descending order of their scores: Lipocalin-2 (Lcn2), Haptoglobin (haptog), RR, CRP, CD163.


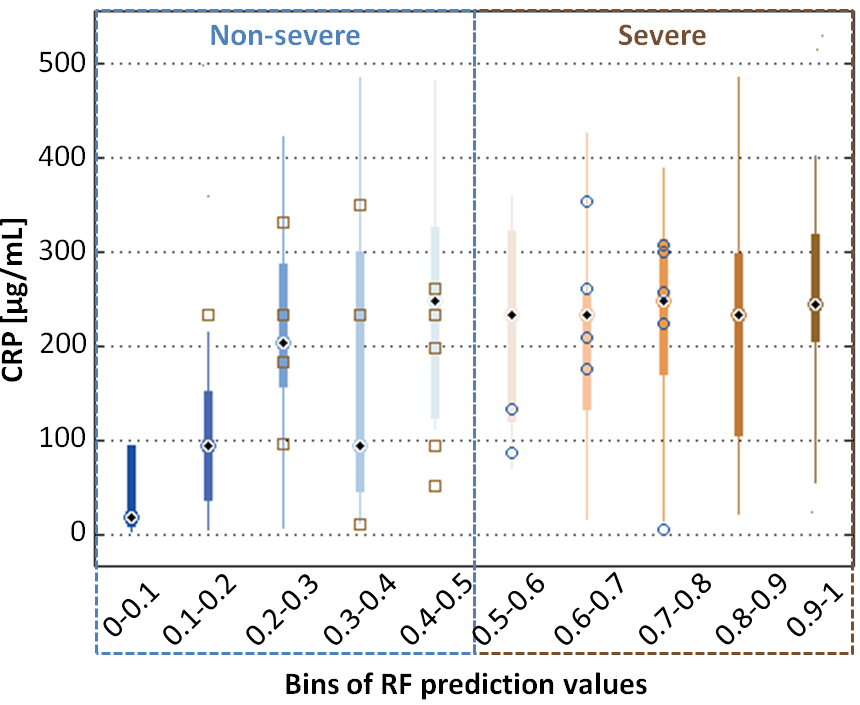


Fig. 4. Distribution of RR values across ten probabilistic groups/bins. Along the x-axis, the range of RF probabilistic predictions was divided into 10 bins, where bin 1 contains cases assigned probabilities between [0,0.1], i.e. 90%-100% certainty of Non-severe Pneumonia, and bin 10 contains cases assigned probabilities between [0.9,1], i.e. 90%-100% certainty of Severe Pneumonia. The number of predicted cases in bins 1-10 were: 29,21,14,23,12,7,19,23,23,28. In each bin, the feature distribution of correctly classified cases is visualised via a boxplot. In each box, the central dot represents the median, the edges are the 25th and the 75th percentiles, and the thin lines extend to the most extreme data points. Misclassified cases in each bin are plotted on top of the boxplot with squares denoting Severe cases and circles - Non-severe ones.

2) Classification: A Gaussian radial basis function kernel within SVM was observed to perform best for the classification problems in this study. However, linear SVM was also attempted with results substantially worse (mean sensitivity and specificity lower by 14% and 12% respectively). This study used Cross-Validation (CV) and Leave-One-Out (LOO) to examine the generalisation of findings to unseen data. Whilst both CV and LOO are popular techniques amongst the machine learning community, it is also possible to perform an alternative split-sample validation. This approach was explored for the first classification problem (Identification of disease), where 10% of the dataset was kept as an out-of-sample test set and the remaining 90% was used or training and identification of the best-performing algorithm. This approach was observed to deliver comparable results: 97.5% (95% CI 94.8% - 100%) sensitivity; 98.8% (95% CI 93.7% - 100%) specificity; 95.1% (95% CI 93.6% - 98.7%). This re-affirmed confidence in the CV and LOO validation techniques.

3) Visualisation: In addition to t-SNE, a number of other techniques were explored for the purposes of visualisation. Neuroscale, as described by Lowe et al. [12], utilises a Radial Basis Function (RBF) neural network to map m feature vectors with N data points to a low-dimensional space. Fiterau et al. have proposed a regression-based technique that uses a nonparametric conditional entropy metric which is minimised to identify a low-dimensional projection. The applicability of both techniques to the pneumonia dataset was investigated. The second technique was unable to deal with the large number of data points and required down-sampling. Both techniques delivered inferior results compared to t-SNE with much greater overlap between classes.

REFERENCES

[1] A. Tsanas, M. Little, and P. McSharry, A methodology for the analysis

of medical data, Chapter 7 in Handbook of Systems and Complexity

in Health. Springer.

[2] H. Stoppiglia, G. Dreyfus, R. Dubois, and Y. Oussar, “Ranking a

random feature for variable and feature selection,” Journal of Machine

Learning Research, vol. 3, pp. 1399–1414, 2003.

[3] R. Tibshirani, “Regression shrinkage and selection via the lasso: A retrospective,” Journal of the Royal Statistical Society.Series B: Statistical Methodology, vol. 73, no. 3, pp. 273–282, 2011.

[4] H. Zou and T. Hastie, “Regularization and variable selection via the elastic net,” Journal of the Royal Statistical Society.Series B: Statistical Methodology, vol. 67, no. 2, pp. 301–320, 2005.

[5] L. Clemmensen, T. Hastie, D. Witten, and B. Ersboll, “Sparse discriminant analysis,” Technometrics, vol. 53, no. 4, pp. 406–413, 2011.

[6] C.-C. Chang and C.-J. Lin, “LIBSVM: A library for support vector

machines,” ACM Transactions on Intelligent Systems and Technology,

vol. 2, pp. 27:1–27:27, 2011, software available at http://www.csie.

ntu.edu.tw/cjlin/libsvm.

[7] T. Hastie, R. Tibshirani, and J. Friedman, The Elements of Statistical Learning: Data Mining, Inference, and Prediction. Springer.

[8] L. Breiman, “Statistical modeling: The two cultures,” Statistical Science, JSTOR, vol. 16.

[9] L. A. Kurgan and K. J. Cios, “Caim discretization algorithm,” IEEE Transactions on Knowledge and Data Engineering, vol. 16, no. 2, pp. 145–153, 2004.

[10] G. F. Cooper, C. F. Aliferis, R. Ambrosino, J. Aronis, B. G. Buchanan, R. Caruana, M. J. Fine, C. Glymour, G. Gordon, B. H. Hanusa, J. E. Janosky, C. Meek, T. Mitchell, T. Richardson, and P. Spirtes, “An evaluation of machine-learning methods for predicting pneumonia mortality,” Artificial Intelligence in Medicine, vol. 9, no. 2, pp. 107– 138, 1997.

[11] G. F. Cooper, V. Abraham, C. F. Aliferis, J. M. Aronis, B. G. Buchanan, R. Caruana, M. J. Fine, J. E. Janosky, G. Livingston, T. Mitchell, S. Monti, and P. Spirtes, “Predicting dire outcomes of patients with community acquired pneumonia,” Journal of Biomedical Informatics, vol. 38, no. 5, pp. 347–366, 2005.

[12] D. Lowe and M. E. Tipping, “Neuroscale: Novel topographic feature extraction using rbf networks,” in Advances in Neural Information Processing Systems, 1997, pp. 543–549.
